# From drug therapy failures to laser therapy victory: A case report and literature review of lupus miliaris disseminatus faciei resolution

**DOI:** 10.1002/ccr3.9370

**Published:** 2024-08-30

**Authors:** Afsaneh Sadeghzadeh Bazargan, Masoumeh Roohaninasab, Hossein Ahmadi Kahjoogh, Saba Baybordi Aghdam, Amirmasoud Taheri, Alireza Jafarzadeh

**Affiliations:** ^1^ Department of Dermatology, Rasool Akram Medical Complex Clinical Research Development Center (RCRDC), School of Medicine Iran University of Medical Sciences (IUMS) Tehran Iran; ^2^ AJA University of Medical Sciences Tehran Iran; ^3^ Mazandaran University of Medical Sciences Sari Iran

**Keywords:** acne agminata, granulomatous dermatosis, LMDF, lupus miliaris disseminatus faciei, pulsed dye laser

## Abstract

**Key Clinical Message:**

Pulsed dye laser (PDL) has proven effective in resolving lupus miliaris disseminatus faciei (LMDF) where drug therapies have failed with a lack of treatment consensus for LMDF, considering early PDL intervention is crucial to achieve resolution without scarring, prevent relapse, and enhance overall treatment outcomes.

**Abstract:**

Lupus miliaris disseminatus faciei (LMDF) is a rare inflammatory and granulomatous dermatologic disease that primarily affects the face. The optimal treatment for LMDF remains controversial, and there is a lack of consensus on the most effective therapy. This case report highlights the successful use of a 595 nm pulsed dye laser (PDL) in the treatment of LMDF following unsuccessful drug therapy. A 28‐year‐old male presented with reddish‐brown eruptions on his face that had persisted for several months. Clinical examination revealed discrete dome‐shaped eruptions in clusters on the central area of the face. Histopathological examination confirmed the diagnosis of LMDF, based on the presence of epithelioid granulomas with central caseous necrosis. Previous treatment with an oral isotretinoin and methotrexate combination also failed to yield satisfactory results. After discontinuing drug therapy, the patient underwent five sessions of PDL treatment. Ten days after the first session, the eruptions began to regress without scarring. Subsequent PDL sessions led to the complete resolution of the eruptions. The patient experienced no relapse during the follow‐up period. This case report suggests that PDL treatment may be an effective option for LMDF, particularly in cases where drug therapy has failed. Early initiation of laser treatment may prevent scarring, minimize the adverse effects associated with drug therapy, and reduce the risk of disease relapse. Further research and controlled trials are needed to establish the efficacy of laser therapy in the treatment of LMDF.

## 1 INTRODUCTION

Lupus miliaris disseminatus faciei (LMDF) is a distinct, uncommon inflammatory and granulomatous dermatologic disease that primarily affects the medial part of the face.^1^, ^2^ However, it has also been reported with extrafacial manifestations.^3^ Despite its name, there is no evidence of acid‐fast bacilli or Mycobacterium tuberculosis DNA in the lesions.^4^ The clinical manifestation of the disease includes discrete reddish‐brown dome‐shaped eruptions in multiple clusters scattered across the face, mainly around the lower eyelids and mid‐face.^5^, ^6^ Histopathology of the lesions consistently demonstrates epithelioid granulomas, mostly with central caseous necrosis.^7^ Although several treatments are suggested in the literature for this disease, there is no unified protocol for therapy. Due to the scarcity of studies, the optimum treatment remains controversial.^8^, ^9^ Laser therapy is known as an effective option in the treatment of various skin diseases, as well as scars caused by inflammatory processes and skin damage.^10^, ^11^ This study presents a case of LMDF with failed drug therapy that achieved a satisfactory result through treatment with a 595 nm pulsed dye laser (PDL).

## 2 CASE HISTORY/EXAMINATION

A 28‐year‐old male was referred to the dermatology clinic with a history of reddish‐brown eruptions on his face that had persisted for 10 months. According to the patient's clinical history, the eruptions began to appear 4 months ago, primarily around the upper lip, nose, and lower eyelids. The lesions were not scaling, exfoliating, pruritic, or painful; however, some of them were crusted. He reported no known triggers for the onset or worsening of the lesions. Three months after the onset of the condition, a physician prescribed oral doxycycline and topical mometasone furoate ointment for 1 month, but no response or improvement was observed.

**FIGURE 1:**
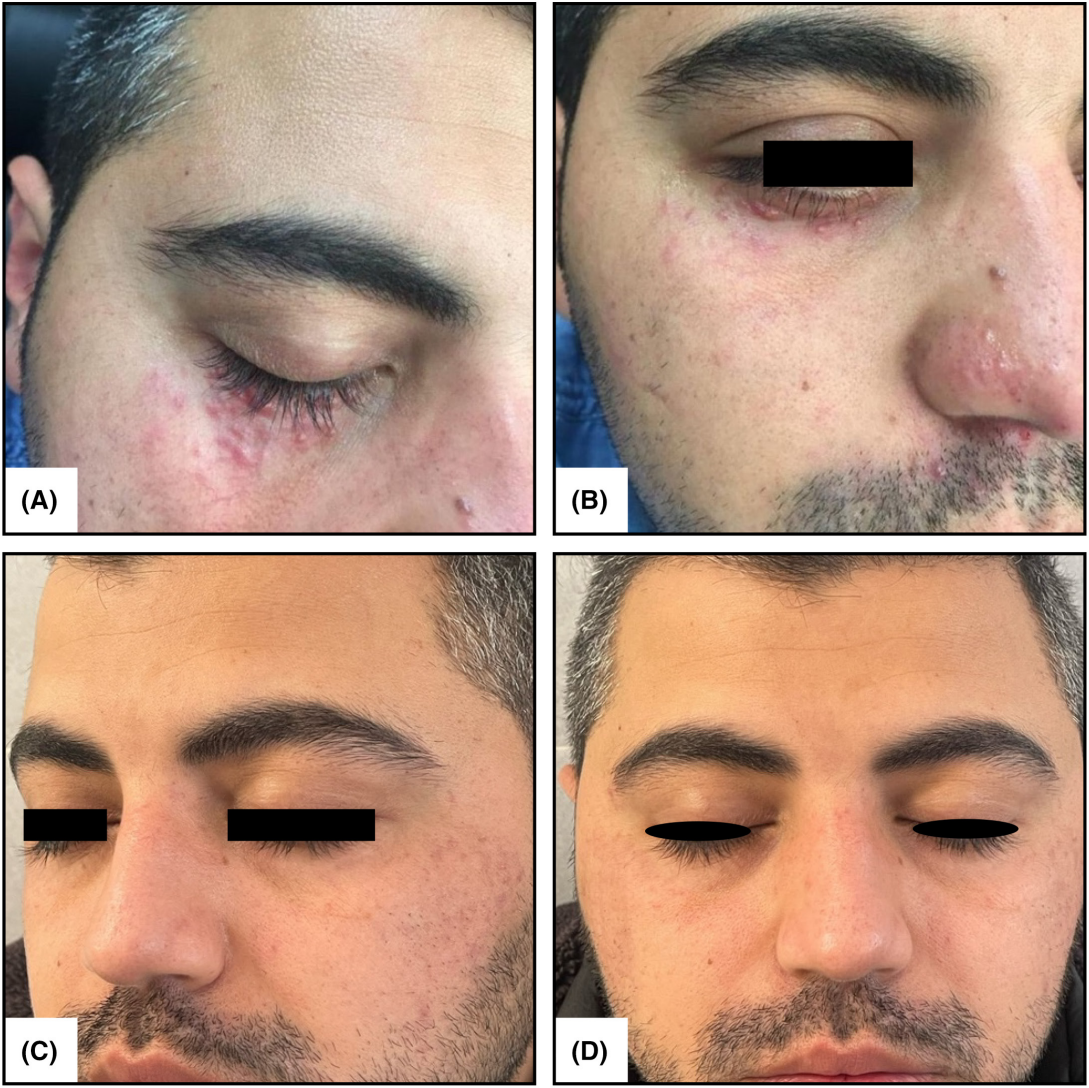
(A and B) discrete dome‐shaped maculopapular eruptions are visible within around lower palpebrae and adjacent to nose. (C and D) Few days After first session of laser treatment, eruptions began to regress without scarring. Scars prior to laser treatment still remained.

During our clinical examination, we observed brown to red discrete maculopapular, acne‐like eruptions in clusters at different stages, ranging from early‐phase lesions to those that had healed with scarring. These lesions measured between 1 to 5 mm and appeared in the central area of his face, including around the lower eyelids, upper lip, and nose (Figure 1A,B).

## 3 METHODS

**FIGURE 2:**
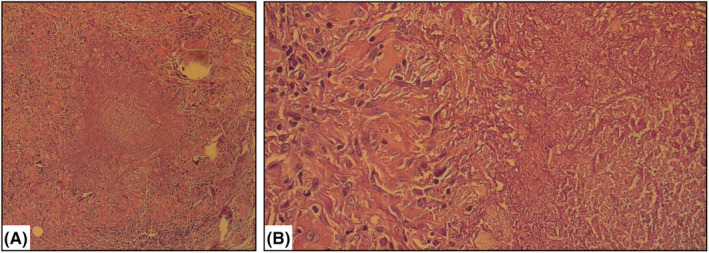
Histopathologic examination revealed Langhans giant cells, or possibly epithelioid and multinucleated giant cell infiltration, along with central caseous necrosis. (A) ×40, (B) ×100.

Histopathological examination of a representative lesion revealed epithelioid multinucleated giant cells, lymphohistiocytic infiltration in the periphery, and caseous necrotic material in the center. Mild infiltration of eosinophils and mast cells was also noted (Figure 2A,B). Furthermore, hyperpigmentation of the basal layer in the overlying epidermis was observed. A thorough microscopic investigation of the biopsied tissue was not consistent with sarcoidal‐type granulomas, mucin deposition, or Demodex mites. This granulomatous tissue pattern, characterized by central caseous necrosis within follicular units, was consistent with LMDF. Additionally, periodic acid–Schiff (PAS) and Ziehl–Neelsen staining were negative for fungal elements and acid‐fast bacilli.

The patient reported no history of previous travel to endemic regions for Mycobacterium tuberculosis or prior exposure to an affected individual. A chest X‐ray was also unremarkable.

After confirming LMDF, oral isotretinoin was administered at a daily dose of 20 mg. However, after 2 months of treatment, the response was unsatisfactory, and liver enzyme levels began to rise. Consequently, the isotretinoin dosage was reduced to 20 mg every other day, and oral methotrexate was added to the regimen at a dosage of 7.5 mg per week for 6 weeks. After 6 weeks of combination therapy with isotretinoin and methotrexate, the results remained unsatisfactory. Therefore, while discontinuing the combination therapy, pulsed dye laser (PDL) treatment was initiated, using a power of 6 J/cm^2^, a pulse width of 0.5 milliseconds, and a wavelength of 595 nanometers.

Ten days after the first laser treatment session, the eruptions began to regress without scarring. PDL therapy was continued for an additional four sessions, with 1 month between each session. At the end of the fifth session, the eruptions were completely resolved without scarring (Figure 1C,D).

## 4 CONCLUSION AND RESULTS

**FIGURE 3:**
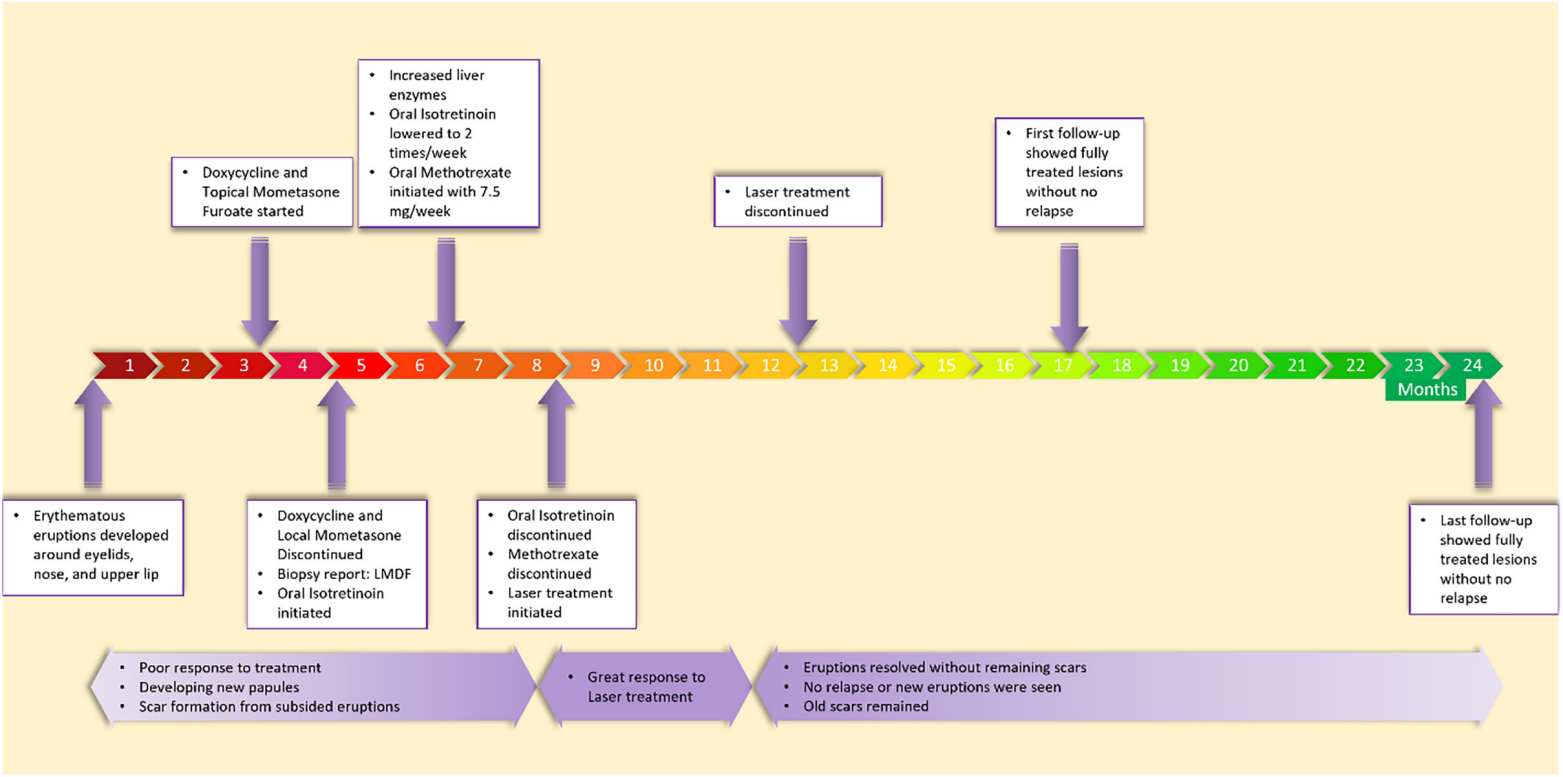
The patient's treatment timeline, indicating the difference between the satisfactory treatment after laser treatment in contrast with the drug therapy.

However, the previous scars, either due to the spontaneous healing of the eruptions or from the drug therapy period, remained. The patient was evaluated five and 12 months after the end of treatment, and no relapse was noted. The timeline of the treatment and progress is presented in Figure 3.

## 5 DISCUSSION

Fox reported the first description of LMDF in 1878 as a disease simulating acne.^12^ As a granulomatous dermatosis, the categorization of LMDF has fluctuated among various granulomatous dermatopathological disorders.^6^ It has been suggested that LMDF is related to Mycobacterium tuberculosis and has been referred to as Lewandowsky's rosacea‐like tuberculid,^13^ micropapular tuberculoid,^14^ or Lupoid rosacea.^15^ However, the presence of acid‐fast bacilli has not been demonstrated in the lesions.^4^, ^7^, ^16^, ^17^ For decades, LMDF was assumed to be a variant of granulomatous rosacea.^4^ In contrast, LMDF's tendency toward self‐regression, the absence of systemic manifestations such as flushing or telangiectasia, and the lack of vasculitis suggest that LMDF is a distinct dermatosis with its own specific features.^2^, ^18^ In the early 2000s, Skowron et al.^1^ described LMDF as a distinctive dermatosis and proposed the acronym “FIGURE” to represent Facial Idiopathic Granulomas with Regressive Evolution. FIGURE captures the disease's characteristics without implying a connection to rosacea, tuberculosis, or other dermatoses.^1^

LMDF or FIGURE naturally tends to regress spontaneously; however, the long disease course often results in residual disfiguring pitted scars, necessitating effective treatment.^19^

Various treatments have been mentioned for this challenging disease, including topical or systemic isotretinoin, antibiotics, corticosteroids, immunomodulators, or combinations of multiple drugs.^20^, ^21^ Intralesional corticosteroid injections have also been reported.^19^ Fewer than 300 LMDF cases have been documented, and few cross‐sectional studies have been conducted, albeit without a control group.^4^, ^17^, ^22^ Consequently, inconsistency in the treatment of LMDF is to be expected. Most suggested treatments have been unsatisfactory for several reasons: first, the disease course is lengthy, and due to the lack of a control group, it is unclear whether regression of the lesions is attributable to the self‐resolving nature of LMDF or the medications used^7^, ^19^, ^22^, ^23^; second, the drugs may cause adverse effects^24^; and third, preventing residual pitted scars is essential. Many reports in the literature that suggest drug therapy as an effective treatment do not mention the status or improvement of residual scars after treatment.^4^, ^6^, ^7^, ^19^ Additionally, the absence of recurring eruptions should also be considered a criterion for successful treatment.

Few studies have reported the implementation of laser treatment for LMDF, as shown in Table 1.^20^, ^24^, ^25^, ^26^ Kang et al.^25^ utilized a carbon dioxide laser in combination with 100% trichloroacetic acid to address the residual scars of LMDF. Before laser treatment, the patient's eruptions had been reduced through drug therapy, including ethambutol, rifampin, and pyrazinamide. Jih et al.^24^ used a 1450 nm diode laser after multiple failures with drug therapies and successfully resolved eruptions without scarring. Beleznay et al.^20^ reported a case of LMDF with poor response to various treatments, including low fluence Nd:YAG 1064‐nm laser and one session of 532‐nm frequency‐doubled Nd:YAG, as well as two sessions of 595‐nm pulsed dye laser (PDL). The patient's eruptions finally regressed without scarring after treatment with a nonablative fractionated 1565‐nm erbium‐doped fiber laser. Six years after Beleznay et al., Ma et al. reported a case of LMDF that was successfully treated with 595‐nm PDL in combination with oral prednisolone, hydroxychloroquine, and isotretinoin.^26^

**TABLE 1:** Features of the last studies on the laser.

Author, Year	Study Size	Prior drug treatment	Efficacy of drug treatment	Laser	Efficacy of laser treatment	Follow up
Jih et al.^24^	1	Topical metronidazole	Ineffective	1450 nanometer diode laser	Resolving rashes without scarring or relapse of eruptions	5 months
		Oral minocycline	Drug‐induced urticaria, Ineffective			
		Oral erythromycin				
		Oral isotretinoin				
		Oral prednisolone				
			Ineffective			
Kang, BK.^25^	1	Minocycline	Little effect	Carbon dioxide laser combined with 100% trichloroacetic	Resolving scars without relapse of eruptions	5 years
		Ethambutol, rifampin and pyrazinamide	Resolving eruptions with pitted scarring			
Beleznay et al.^20^	1	Oral doxycycline	Ineffective	Three sessions of low fluence Nd: YAG 1064 nm laser	Ineffective	
		Oral tetracycline amoxicillin/clavulanate			Ineffective	
					Ineffective	
		Short course of systemic corticosteroids				
					Resolving rashes without scarring or relapse of eruptions	
				Two sessions of 595 nm pulsed dye laser (PDL)		
		Oral itraconazole				
				One session of 532 nm frequency‐doubled Nd:YAG		
		Oral isotretinoin				
		Topical metronidazole				
		Topical isotretinoin				
		Topical pimecrolimus		Nonablative fractionated 1565 nm erbium‐doped fiber laser		
		Topical permethrin				
		Intralesional triamcinolone acetonide				
Ma et al.,^26^	1	Topical erythromycin	Alleviating rashes	595‐nm pulsed‐dye laser combined with Oral prednisolone, hydroxychloroquine, and isotretinoin	Resolving rashes without scarring or relapse of eruptions	5 months
		Topical traditional Chinese medicine				
		Oral prednisolone, hydroxychloroquine, and isotretinoin				

The results of the current study are consistent with those of Ma et al.^26^; however, in our case, the drugs were discontinued first, followed by the initiation of PDL sessions without combination therapy.

Despite the limited treatment options for LMDF, our results, along with previous findings, suggest that laser therapy (595‐nm PDL or 1450‐nm diode laser) may be more effective than multiple courses of drug therapy. Although controlled trials with larger sample sizes are needed to establish the efficacy of treatments, we recommend initiating laser therapy earlier to alleviate eruptions without scarring, avoid multiple drug failures and their side effects, prevent disfiguring scars, and reduce the risk of disease relapse.

## AUTHOR CONTRIBUTIONS

**Afsaneh Sadeghzadeh Bazargan:** Conceptualization. **Masoumeh Roohaninasab:** Project administration. **Hossein Ahmadi Kahjoogh:** Investigation; methodology. **Saba Baybordi Aghdam:** Data curation. **Amirmasoud Taheri:** Writing – original draft. **Alireza Jafarzadeh:** Data curation; writing – original draft.

## FUNDING INFORMATION

None.

## CONFLICT OF INTEREST STATEMENT

The authors declare no conflicts of interest.

## ETHICS STATEMENT

The researchers were committed and adhered to the principles of the Helsinki Convention and the Ethics Committee of the Iran University of Medical Sciences in all stages.

## CONSENT

Written informed consent was obtained from the patient to publish this report in accordance with the journal's patient consent policy.

## TRANSPARENCY DECLARATION

Authors declare that the manuscript is an honest, accurate, and transparent. No important aspect of the study is omitted.

## Data Availability

All data produced in the present study are available upon reasonable request to the authors.
